# Brain Metabolism in Health and Neurodegeneration: The iInterplay Among Neurons and Astrocytes

**DOI:** 10.3390/cells13201714

**Published:** 2024-10-17

**Authors:** Polina Shichkova, Jay S. Coggan, Henry Markram, Daniel Keller

**Affiliations:** 1 Blue Brain Project, École Polytechnique Fédérale de Lausanne, 1202 Geneva, Switzerland; 2 Laboratory of Neural Microcircuitry, Brain Mind Institute, École Polytechnique Fédérale de Lausanne, 1015 Lausanne, Switzerland

**Keywords:** astrocyte, brain energy metabolism, brain aging, neurodegeneration, brain glucose, brain lactate, brain glycogen, brain ketones

## Abstract

The regulation of energy in the brain has garnered substantial attention in recent years due to its significant implications in various disorders and aging. The brain’s energy metabolism is a dynamic and tightly regulated network that balances energy demand and supply by engaging complementary molecular pathways. The crosstalk among these pathways enables the system to switch its preferred fuel source based on substrate availability, activity levels, and cell state-related factors such as redox balance. Brain energy production relies on multi-cellular cooperation and is continuously supplied by fuel from the blood due to limited internal energy stores. Astrocytes, which interface with neurons and blood vessels, play a crucial role in coordinating the brain’s metabolic activity, and their dysfunction can have detrimental effects on brain health. This review characterizes the major energy substrates (glucose, lactate, glycogen, ketones and lipids) in astrocyte metabolism and their role in brain health, focusing on recent developments in the field.

## 1. Introduction

Brain function is accompanied by significant energy costs, which can quickly vary depending on neuronal activation. Dependence upon a continuous supply of energy substrates such as glucose, lactate, fatty acids and ketones, is critical to brain function because the brain has limited internal energy stores and experiences large variation in energy demand as a result of activity. Consequently, the blood supply of these energy substrates and the removal of by-products need to be well-coordinated with the regime of neuronal activity. The blood–brain barrier (BBB) and neurovascular coupling (NVC) are two distinct features of the brain vasculature that underlie metabolic homeostasis in the brain [1,2]. The uptake and use of nutrients for brain function is regulated by biochemical reactions and signaling processes spread across different cell types. Astrocytes, physically located between neurons and blood vessels, are actively involved in NVC, in part by modulating the availability of energy substrates and oxygen [3,4,5,6,7,8] via vasodilation [9,10]. Accordingly, a significant part of the astrocytic proteome (including electron transport chain proteins) is found in the perivascular endfeet [11]. Astrocytes not only serve as functional mediators of NVC but also participate in formation and maintenance of the BBB, as reviewed elsewhere [12]. The functional unit of brain energy metabolism is commonly considered to be the neuro–glia–vasculature (NGV) ensemble [13]. This unit is often modeled as one neuron, one astrocyte and a representation of the local microvasculature [14].

The relationship between mammalian neuronal signaling, energy demand and supply, governed by the collaboration of different types of cells, requires specialization of some functions in those cells. For example, there are many functionally important differences in metabolic features between astrocytes and neurons which likely play a role in their joint energy management (Table 1). More precise measurement of metabolic responses to neuronal activation is needed to provide the detailed mechanisms which make predictive energy allocation possible [15]. This could enable novel treatment of brain disorders, such as neurodegenerative diseases, because energy metabolism malfunction is implicated in the early stages of disease development [16].

As brain energy metabolism is managed by the intricate cooperation of different cell types, disturbances in any component or their interaction ultimately affect brain function. Perturbations in brain energy metabolism commonly manifest in aging, for example, which is considered one of the key risk factors of neurodegenerative disorders [19], even though the etiology is not completely understood, and these diseases show a range of clinical manifestations. Changes in brain metabolism are found years before cognitive decline in aging and Alzheimer’s disease (AD) [62], and the effects are observed at multiple scales of brain organization, as described below.

At the full brain scale, metabolic aging is tied to the global decrease in cerebral blood flow, associated with reductions of oxygen and nutrient supplies [63] and glucose metabolism [64]. An additional link between an observed decrease in brain volume [65] and deficient energy supply in aging can, therefore, be suggested. Brain regions show different susceptibility to aging and neurodegenerative diseases [66], which can at least in part be related to energy metabolism. Vulnerable regions are rich in synapses, which imply high energy burden and risks of degeneration in case of mismatching supply [67]. Many such regions possess long-range axons, which are prone to energy metabolism deficits [68]. Astrocytes are also highly heterogeneous, and pathological changes in their functions contribute to selective vulnerability of various brain regions to degeneration [69,70]. Furthermore, given metabolic modulation of network synchrony [71], a causal relation between metabolism and subsequent cognitive decline can be hypothesized.

At the level of intercellular crosstalk, glia-mediated coordination of neuronal activity and blood flow is affected by aging [72]. Vascular impairments are associated with both AD pathology [73] and AD-independent apolipoprotein E4 (APOE4)-associated cognitive decline [74]. Metabolic cooperation of astrocytes with neurons decreases in AD, as shown by downregulated expression of genes responsible for fatty acid transport, storage, oxidation and related detoxification of reactive oxygen species [75]. Yet gliotransmission is enhanced in AD, while it is largely unaffected in healthy aging [76]. While changes in gliotransmission in healthy aging and AD are not fully understood, one explanation for the differences could be compensation for disease processes, and this might offer a clue about a particular deficiency in AD. There are many mechanisms for pathological changes in regulatory and signaling pathways, such as Ca^2+^-signaling. Furthermore, pathologically enhanced functions such as gliotransmission may be associated with multicellular communication.

**Figure 1:**
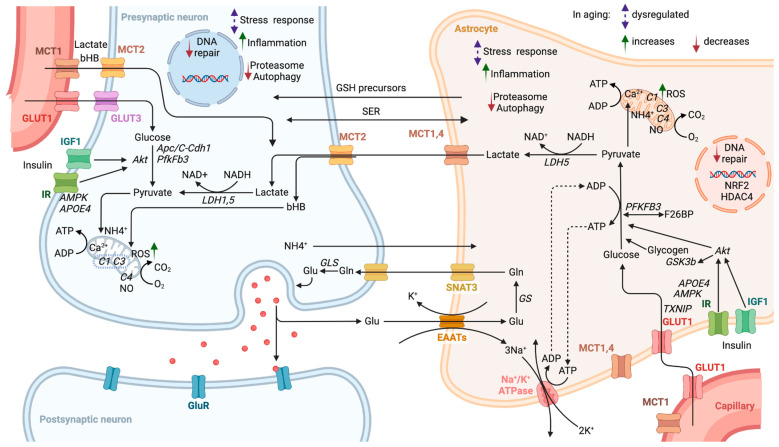
Metabolism regulation and aging effects in NGV crosstalk. The key metabolic pathways of the main brain energy sources, their regulation, metabolic cooperation and aging-related changes in the NGV. Abbreviations: MCT1—monocarboxylate transporter 1; bHB—β-hydroxybutyrate, GLUT1—glucose transporter 1; MCT2—monocarboxylate transporter 2; GLUT3—glucose transporter 3; IGF1—insulin-like growth factor 1; IR—insulin receptor; AMPK—adenosine monophosphate (AMP)-activated protein kinase; APOE4—apolipoprotein E4; DNA—deoxyribonucleic acid; Apc/C-Cdh1—anaphase-promoting complex/cyclosome Cdh1; PfkFb3—6-phosphofructo-2-kinase/fructose-2,6-biphosphatase 3; NAD^+^—oxidized nicotinamide adenine dinucleotide; NADH—reduced nicotinamide adenine dinucleotide; LDH1,5—lactate dehydrogenases 1,5; NH_4_^+^—ammonium; ROS—reactive oxygen species; O_2_—oxygen; CO_2_—carbon dioxide; NO—nitric oxide; C1—complex 1 of the electron transport chain; C3—complex 3 of the electron transport chain; C4—complex 4 of the electron transport chain; ADP—adenosine diphosphate; ATP—adenosine triphosphate; Glu—glutamate; Gln—glutamine; GLS—glutaminase; GluR—glutamate receptors; GS—glutamine synthetase; SNAT3—sodium-coupled neutral amino acid transporter 3; EAATs—excitatory amino acid transporters; GSH—glutathione; SER—serine; Ca^2+^—calcium; LDH5—lactate dehydrogenases5; NRF2—nuclear factor erythroid 2–related factor 2; HDAC4—histone deacetylase 4; F26BP—fructose 2,6-bisphosphate; GSK3b—glycogen Synthase Kinase 3 β; Akt—protein kinase B (Akt kinase); TXNIP—thioredoxin-interacting protein; MCT1,4—monocarboxylate transporters 1,4; Na^+^—sodium; K^+^—potassium; Na^+^/K^+^ ATPase—sodium-potassium pump. Figure created in Biorender.

At the scale of single cells, every cell type in the brain is affected by aging and associated disorders, including astrocytes [77,78], oligodendrocytes [79], microglia [80] and ultimately neurons [81]. Within each cell, at the molecular level of detail, hallmarks of aging [82,83] and neurodegeneration [84] include pathways involved in mitochondrial function, oxidative stress response, inflammation, autophagy and damage repair [85]. Accordingly, brain energetics have become a target of preclinical and clinical studies [86]. The key metabolic pathways of the main brain energy sources, their regulation, metabolic cooperation and aging-related changes in the NGV ensemble are highlighted in Figure 1. Aging-associated changes are system-wide and affect various processes from glucose transport to deoxyribonucleic acid (DNA) repair and gene expression. However, causes for the transition from the aging brain to neurodegenerative conditions remain unclear. Accordingly, better understanding of the differences between aging and neurodegeneration can help prevent pathological transition and potentially alleviate the disease. Towards this goal, we characterized the metabolic function of astrocytes in healthy aging and neurodegeneration.

## 2. Glucose: Caveats of the Default Brain Fuel

Neurons, and synaptic transmission in particular, account for most of the ATP expense in the brain [87]. This energy is mostly supplied from the catabolism of glucose [88]. Aspects of this central dogma of brain metabolism, such as in vivo measurements of the glucose analog uptake rate [33], expression of key enzymes [33] and transporters [89] mediating glucose metabolism, as well as their kinetics [89,90,91], have been assessed over the years by an enormous number of studies and applied in practice by positron emission tomography (PET) and functional magnetic resonance imaging (fMRI) methods [92]. In both laboratory and clinical settings, glucose (via PET imaging) and oxygen consumption (via fMRI) are used as a proxy for neuronal activity. This is just one of the reasons why understanding the decomposition of glucose utilization by different cell types and even subcellular compartments is of particular practical importance for diagnostics of abnormal brain function.

The astrocytic share of brain energy metabolism has not traditionally received as much attention as that of neurons, but it has recently been the subject of renewed interest [93,94,95]. Even so, the synapse is still responsible for the most energy consumption. Neurons take up more glucose and have higher hexokinase expression than astrocytes [33], which bolsters the case for considering glucose as the preferential neuronal energy fuel. However, there is a branching point of glycolysis and PPP downstream of hexokinase, which points to a role for glucose in redox state maintenance via PPP. The glycolytic rate in neurons is lower than in astrocytes, which is at least partially explained by degradation of the positive regulator of glycolysis PFKFB3 by the anaphase-promoting complex/cyclosome (APC/C) Cdh1 [32]. Interestingly, lactate regulation of APC/C has been recently discovered in rapidly dividing cells [96]. PFKFB3 is active in astrocytes, however, and likely contributes to the observation that glycolysis is capable of maintaining the viability of adult respiration-deficient astrocytes a year after induced impairment of mitochondrial cytochrome c oxidase assembly in mice [97]. Notwithstanding this astrocytic capability of surviving on glycolysis alone for extended periods, oxidation occurs in this cell type under normal conditions. Moreover, mitochondrial metabolism in astrocytes is linked to brain metabolism regulation and redox balance [98], as various TCA cycle intermediates possess signaling functions [99]. Furthermore, some glial cells can sense mitochondrial stress via small vesicle signaling to neurons to alleviate the protein aggregation often found in brain aging and neurodegenerative diseases [100].

Neurons require oxidative phosphorylation for their high energy output. However, oxidative processes are linked to reactive oxygen species (ROS) production [101]. Neuronal complex I of the electron transport chain is mostly assembled into supercomplexes, contrary to the high fraction of free complex I in astrocytes, which correlates with a higher rate of ROS generation by astrocytes [47]. Such ROS production is not just deleterious but also supports a basal level of Nrf2 (master regulator of antioxidant gene expression) activity which further regulates neuronal function via metabolic cooperation of these cells [102,103]. Furthermore, a mechanism for shuttling glucose from glia to neurons with its subsequent utilization in the PPP, which plays an important role in redox state maintenance, was recently linked to long-term memory formation in *Drosophila* [104].

Interestingly, active regulation of circulating metabolite concentration has only been observed for glucose (involving insulin) and not for the other tested molecules (3-hydroxybutyrate, citrate and several amino acids) in a recent study by Li et al. [105,106]. However, lactate, glutamine and free fatty acids were not measured in that study due to technical limitations. There may be even more intricate mechanisms that regulate levels of circulating metabolites other than glucose. It can be speculated that increased levels of such metabolites serve as a signal to use a surplus for anabolic processes and internal resource accumulation. However, the lack of active regulation of circulating metabolites may merely be a consequence of significantly dysregulated conditions not normally existing. This hypothesis is supported by the observations that disturbed circulating metabolite homeostasis is associated with diseases [107], one of the most known and common of which is diabetes [108].

### Glucose in Aging and Neurodegeneration: More Than a Fuel

A number of studies show beneficial effects of different diets on aging and neurodegeneration-associated changes [109,110,111]. However, the underlying mechanisms of action largely remain elusive. A systematic understanding of all non-energy functions of brain fuels can help separate between molecule-specific effects and those of the fuel-independent energy status of the cell.

Glucose uptake and utilization is lower in aging brains and is accompanied by significant metabolic imbalance [112,113]. Decreased glucose levels correlate with decreased ATP concentration in the aged *Drosophila* brain, a condition ameliorated by the overexpression of the glucose transporters [114]. Furthermore, changes in oxidative glucose metabolism with aging are not proportional to those of glycolysis, meaning that aerobic glycolysis is predominantly decreased in aging [115]. Disruptions in aerobic glycolysis are also implicated in AD pathology [116].

Aging-associated vascular degeneration lowers glucose supply and induces glucose hypometabolism, resulting in the activation of AMP-activated protein kinase (AMPK) if the ATP/AMP ratio is not maintained by other energy producing pathways. AMPK would normally activate energy production, and one of its targets is the insulin receptor (IR), whose trafficking interaction with insulin are in turn impaired by the APOE4 human apolipoprotein isoform [117], a well-known risk factor for AD. A number of recent studies describe AD as “type 3 diabetes”, attributing a significant role to the brain glucose metabolism impairment in the etiology of this disease [118,119,120]. Contradictory evidence, however, does exist with regard to brain insulin sensitivity or resistance in aging as reviewed by Case and colleagues [121].

There are many mechanisms implicating the role of glucose in AD. Some of these include mitochondrial calcium dysregulation and inflammation [122]. Glucose metabolism is also directly linked to synthesis of products whose levels decline in aging, such as the neurotransmitters glutamate and gamma-aminobutyric acid (GABA) [123,124,125] and the important antioxidant glutathione [126]. Impaired neuronal glycolysis has been identified as a link between peripheral hyperinsulinemia triggering neuronal insulin resistance and subsequent cell-cycle dysregulation promoting neurodegeneration in aging [127]. In astrocytes, loss of insulin signaling has been linked to not only lower ATP production but also impairment of the astrocytic uptake of β-amyloid in AD [128]. The enzyme Akt kinase is downstream of insulin signaling, and among its numerous targets are hexokinase, PFKFB3 and glycogen synthase kinase-3 β (GSK3β)—which have all been implicated in neurodegenerative processes [129,130,131,132,133]. Glucose is also one of the key elementary building blocks for the ubiquitous glycosylation of macromolecules in the brain, which shows region specificity and age-dependent patterns [134]. Changes in glycosylation are observed in neurodegenerative disorders, such as AD and PD [135,136]. Glucose is also known to regulate autophagy [137,138], the process of cellular “self-eating”. Autophagy’s role is not only in the degradation of old and damaged cellular components but also in the creation of new building blocks and energy substrates for the cells. It is, therefore, tightly linked to the energy balance and is a hallmark of aging [83]. Some of these mechanisms within the context of the NGV are illustrated in Figure 1.

Altogether, while aging is associated with numerous changes in brain glucose metabolism, progressive decline manifests in the perturbation of different glucose-dependent functions of the brain. Better understanding of glucose-exclusive components of the system and those able to rely on complimentary energy sources, as well as regulatory processes directing glucose where it is the most needed, can help slow down the transition between healthy aging and age-associated brain disorders.

## 3. Role of Lactate in Normal Brain Function

Lactate has been known for many decades as a metabolic by-product [139,140,141,142,143,144] and has been mostly studied in muscle. The function of lactate as an energy substrate in the brain has been addressed by a growing number of studies starting from (to our knowledge) the work of McIlwain in 1953 [145]. The early development of lactate as a brain fuel concept was reviewed by Pellerin in 2003 [146]. However, the importance of lactate as an energy substrate, as well as an astrocyte-to-neuron lactate shuttle (ANLS) theory [147], has provoked a long-standing debate and motivated numerous studies starting from Chih et al. [17] and further discussed by others [148,149,150,151,152,153,154,155]. At present, this topic still continues to engage research on various related biological aspects and motivate new experimental measurements [156,157,158,159,160,161,162]. Conditions favoring one or the other direction of lactate shuttling have been assessed computationally, suggesting that the dynamics of lactate transport between astrocytes and interstitium play a key role [163].

The distribution of lactate dehydrogenase (LDH) isoform expression in neurons and particularly in energy demanding synaptosomes differs from that of astrocytes as analyzed by O’Brien et al. [38]. The isoenzyme which favors production of pyruvate from lactate (LDH1) is predominant in synaptosomes, while other isoforms are present in both neurons and astrocytes [17,38,164]. Interestingly, almost one-third of synapses do not contact astrocytic processes [165]. Use of lactate as an energy substrate by either cell (or even part of the cell) is directly linked to the oxidized/reduced nicotinamide adenine dinucleotide (NAD^+^/NADH) state and may be involved in neuron–astrocyte redox coupling [166]. Furthermore, astrocytic redox imbalance in superoxide dismutase 2 (SOD2)-deficient mice leads to an accelerated aging and hypometabolic phenotype [167]. Novel roles of lactate in the brain have recently been identified, such as the requirement of the transfer of glycogen-derived lactate from astrocytes to neurons for long-term memory formation [168,169] and the role of lactate as a signaling molecule [170]. Taken together, the considerations above reflect a multifaceted role of lactate in the ANLS, as previously reviewed by Tang [171].

A lactate concentration gradient from astrocytes to neurons in rodents has been observed to be necessary [156] for proper function of monocarboxylate transporter-mediated export of lactate from astrocytes to extracellular space and ensuing uptake by neurons. The emergence of the lactate gradient can be explained by more efficient proton-coupled lactate/pyruvate transport in astrocytes than in neurons [159]. However, which combination of mechanisms and conditions is sufficient for ANLS remains unclear. Further aspects of the ANLS question are detailed by Dienel with regard to glycolytic and lactate release rates, oxygen-to-glucose index (OGI), difference between cultured cell systems and the brain in vivo [172]. The differences between the observations and predicted behavior highlight potential gaps in our understanding of the underlying mechanisms at the multiscale level, where cells and tissue signals are ultimately the result of numerous reactions, signaling interactions, transport events and diffusion at the subcellular level. ANLS may complement internal neuronal glucose catabolism to distribute the burden and compensate for mismatch in expected and available energy resources. The signaling function of lactate could be part of the regulatory mechanism orchestrating energy demand and supply spatially and temporally.

Recently, Muraleedharan et al. [161] have shown that the energy balance-sensing AMPK regulates astrocytic glycolysis and ANLS. Neuronal survival appeared to be dependent on AMPK activity in the astroglia but not in the neurons themselves. This finding further lends credence to the idea that astrocyte-derived lactate is needed for neuronal function. If the neurons in co-culture with astrocytes survived after the deletion of AMPK, which is known as “the master regulator of energy metabolism”, then ANLS is potentially sufficient for supporting neuronal function, at least under some conditions. However, AMPK has multiple targets in the cell [173] which leaves the potential for an intricate interplay of downstream intracellular pathways.

Lactate signaling through ATP-sensitive potassium channels results in a firing frequency increase [174]. Furthermore, it attenuates synaptic transmission and increases oxygen consumption in neuronal firing states of high-energy use, but use of lactate as the only fuel has been shown to be less efficient than glucose in supporting fast network oscillations [160]. Synaptic plasticity is associated with metabolic plasticity as evaluated from the extracellular lactate dynamics in response to induced long-term potentiation [175]. Some recent studies associate lactate export from the brain by the glymphatic system with the sleep–wake cycle [176]. Other observations show that a state of arousal evokes β-adrenergic signaling regulated, glycogen-derived lactate release from astrocytes [162]. All these multifaceted signaling functions of lactate are potentially only the first glimpse of the expanding complex role of lactate as a messenger molecule.

There are less-studied roles of lactate shuttling as well. The neuromodulatory function of lactate in decreasing synaptic activity via action on hydroxycarboxylic acid receptor 1 (HCAR1) has been suggested by some recent studies [31,177], and it is relevant for developing novel therapies against seizures. HCAR1 has also been implicated in regulating tissue regeneration after hypoxia-ischemia in newborns [178]. Another role of ANLS has been found in antioxidant defense. One of the harmful effects of ROS is lipid peroxidation. However, the formation of lipid droplets helps to counteract this effect [179]. ANLS fuels neuronal lipid production in response to the increase in ROS levels. There is subsequent lipid transport to astrocytes [180] and formation of astrocytic lipid droplets [181]. This “neuronal lipid outsourcing” permits the mitigation of harmful ROS effects on the neurons. Furthermore, APOE4 impairs this neuron–astrocyte shuttle of lipids, which may be a mechanism of neurodegeneration [181].

Our understanding of the role of lactate in the brain and other tissues is currently undergoing a paradigm shift [158,182]. Lactate uncouples mitochondrial oxidation from glycolysis and together with pyruvate it balances the NADH/NAD^+^ redox state [183]. At the same time, levels of this universal metabolite are tightly regulated at the organism scale, balancing the activities of glycolysis and TCA by mechanisms which are not yet fully clear. Furthermore, the uniqueness of lactate as a universal TCA substrate has been demonstrated in a recent quantitative fluxomics study by Hui et al. [184]. The same study also highlighted stable preferences in energy substrates across many tissues, confirming that the brain mainly uses glucose with significant contribution from lactate. Contrary to the concept of a ketosis shift in brain metabolism [185,186], the authors show that carbohydrate preference remains even on a ketogenic diet, where liver and kidney support circulate glucose levels by converting glycerol into glucose. Observing lactate from the whole organism scale and recalling the physical exercise during which it was first studied, one could consider lactate as a mediator of the beneficial effects of physical activity on the brain, offering neuroprotective properties [187]. Furthermore, a specific therapy (lactate threshold training) relying on lactate-mediated beneficial effects of physical exercise has been shown to improve the quality of life of patients with multiple sclerosis [188].

### Brain Lactate in Aging and Neurodegeneration

Discordant evidence exists regarding the role of brain lactate in aging and neurodegeneration. Brain lactate levels were found to be twofold higher in aging due to the shift in ratio of LDH isoforms expression [189], but these results have been debated [190]. Recently, higher lactate levels were also reported in AD patients than in cognitively unimpaired participants of the study [191], but an earlier study with astrocytes from induced pluripotent stem cells derived from AD patients showed a reduction in astrocytic lactate secretion [192]. Interestingly, a decrease in the lactate shuttle has been reported among the other aging-associated changes of astrocyte function [193]. Lowered lactate levels were also observed in amyotrophic lateral sclerosis (ALS) [194]. Increased levels of lactate in aged astrocytes have been suggested as acting via nuclear factor kappa-light-chain-enhancer of activated B cells (NF-κB) signaling to downregulate neuronal gonadotropin-releasing hormone (GnRH-I), which regulates reproduction and is known to be lowered in aging [195]. These divergent observations call for better understanding of the relation between lactate levels in brain cell types and its transport in different ages and diseased conditions.

Chronic inflammation associated with aging and neurodegeneration has been shown to increase ANLS in cell co-cultures, while neuronal oxidation of lactate was decreased as inferred from decreased NADH/NAD^+^ levels [196]. A recent study in *Drosophila* showed that inflammatory cytokines from the gut metabolically reprogram glial cells to increase lactate and lipid transport in the brain, and this is related to olfactory decline in aging [197]. However, other studies have shown a decrease of astrocytic lactate production and metabolic support to neurons as a result of proinflammatory cytokines-driven astrocyte remodeling [198]. Histone lactylation has recently been demonstrated to regulate transcription [199] and has also been implicated in AD [200].

## 4. The Purpose of Glycogen Storage

The brain’s internal energy stores are limited [92]. Glycogen is considered by a number of studies to be such a source [23,149]. It is predominantly located in astrocytes, which were even classified into two types based on the amount of glycogen identified in single-cell analysis [201]. However, glycogen has also been found in neurons in some pathophysiological conditions [24,202]. Furthermore, glycogen phosphorylase expression in neurons has also been observed [26], and sex-associated differences in glycogen phosphorylase were observed in hypothalamic astrocytes [203].

Astrocytes support neurons by glycogen-derived energy resources during high energy demand periods [204], in neurodegeneration-related metabolic stress [205] and via salvianolic acid B-mediated neuroprotection after ischemic stroke [206]. There are two main theories as to how astrocytic glycogen can serve as an energy source for neurons: via ANLS [207,208] or by so-called glucose sparing by glycogenolysis [209]. Meanwhile, it has been noted that glucose supply is unlikely to be a rate-limiting step for the energy production under physiological conditions, in which glycogen mobilization still takes place upon increased energy demands [210]. Glycogen has been shown to play a role as an energy source in learning [211,212], working and short-term memories [213]. Its turnover and accumulation has also been studied in sleep/wake cycles [214].

Along with the multipurpose function of many other molecules, energy storage is not the only designated function of glycogen. The thermodynamic function of glycogen metabolism in phosphate buffering for maximization of energy yield has also been suggested as an explanation for many phenomena [210]. Furthermore, Sun and colleagues [215] demonstrated the role of glycogen as a glucosamine pool for protein glycosylation and impairment in glycogen storage diseases. If brain glycogen is more than a static reservoir for the supply of glucose, how is this multipurpose store regulated to serve all different functions? Glycogen mobilization is regulated by cyclic adenosine monophosphate (cAMP) [216] and calcium [217,218], but to date there is no complete understanding of these mechanisms’ contributions.

### Brain Glycogen in Aging and Neurodegeneration

There are contradictory views on the function of glycogen in neurons in health and disease. For example, glycogen metabolism has been attributed a role in proteolytic processes and autophagy [27,219]. Defects of glycogen metabolism are implicated in Lafora disease, seizures and neurodegeneration [220,221]. Furthermore, glycogen synthase has been found in specific granular formations that are produced by astrocytes mostly in aging, in neurodegeneration and involved in waste removal from the brain [222].

Glycogen metabolism enzymes have been found at elevated concentrations in aging hippocampal neurons, while in the young brain they are expressed in the astrocytes [28]. This observed change in glycogen metabolism may indicate a decline in neuronal reliance on astrocyte-derived lactate in aging [28]. Furthermore, distribution of glycogen in astrocytes changes with age: glycogen is organized in patches in young but not aged mice, while observed glycogen levels are similar in both age groups [20]. Another recent study found a metabolic shift from aerobic glycolysis to oxidative phosphorylation in aging astrocytes, which reduces their ability to export lactate [223]. Glycogenolysis can be triggered by the release of norepinephrine (NE) by activation of the locus coeruleus (LC) network which signals an increase in attention or increased regional brain activity. Dysfunction of LC-NE-related glycogen mobilization has accordingly been implicated in many degenerative diseases associated with aging [224,225]. Various other suggested roles of glycogen in brain disorders have been reviewed by Bak et al. [22].

## 5. Ketones and Lipids in Normal Metabolism

Ketone bodies can be oxidized as an energy source in the brain [226]. The use of ketone bodies in the brain is promoted by deacetylating 3-oxoacid CoA-transferase (OXCT) and acetyl-CoA acetyltransferase (ACAT1), by a member of the sirtuin family of deacetylases, NAD-dependent deacetylase sirtuin-3 (SIRT3) [227], known for its energy-metabolism and reactive oxygen species management, as well as its involvement in aging. While ketone bodies can be supplied by blood circulation, they can also be synthesized by astrocytes and released into the extracellular space from where neurons can take them up [228]. As in the case of glycogen, a glucose-sparing function has been recently suggested for ketone bodies [229]. Analogous to ANLS, the neuroprotective astrocytic ketone bodies shuttle became a point of interest in some studies [230,231]. Moreover, elevated lactate levels have been reported in ketosis [232,233]. Consistent with shuttling between two cell types, differential effects of the ketogenic diet on transcription in neurons (with upregulated expression of genes attributed to multiple pathways) and astrocytes (with both up and down expression changes depending on pathways) have recently been reported [234].

As for other substrates reviewed above, the supply of energy is not the only function of ketone bodies [235,236]. β-hydroxybutyrate (the principal ketone body) and products of its metabolism exert signaling functions on metabolism and gene expression, in turn affecting mechanisms implicated in aging, neurodegenerative disorders and energy deprivation [237,238,239]. However, controversies still exist, especially with regard to extrahepatic ketogenesis and at least partially related to the reversibility of succinyl-CoA:3-oxoacid-CoA transferase (SCOT/OXCT1) and thiolase [240].

β-hydroxybutyrate inhibits astrocytic glucose consumption and glycolysis, but this metabolic downregulation is paralleled by activation of mitochondrial pyruvate metabolism [241]. A recent study in starving *Drosophila* [242,243] showed an AMPK-regulated contribution of the glia-produced ketones in memory formation. Another ketone body, acetoacetate, non-enzymatically reacts with a toxic by-product of glycolysis, methylglyoxal, thereby buffering its bioavailability [244]. Details of ketone body metabolism and the effects of the ketogenic diet are available in various literature reviews [236,245].

Direct fatty acid oxidation in the brain (β-oxidation pathways) has long been considered as not favored due to the production of the dangerous superoxide ion, as well as a higher use of oxygen and a lower rate of ATP generation as compared to glucose metabolism [246]. However, these effects are mitigated by astrocytes, where fatty acid oxidation provides benefits to the neurons [180,247] through well-coordinated neurometabolic coupling.

### Brain Ketones and Lipids in Aging and Neurodegeneration

Lipids provide one of the major sources of energy for astrocytes, but both ketone bodies and lipid metabolites predominantly feed into the TCA cycle at the acetyl-coenzyme A (AcCoA) entry point, so it might be difficult to determine their differential effects [59]. Studies indicate that brain lipid peroxidation is not a significant feature of normal aging but rather an indicator of pathological dependence on lipid β-oxidation with ROS production comorbidity [248]. In fact, metabolites of lipid peroxidation (LPO) are present in the cerebrospinal fluid (CSF) of AD patients in the prodromal phase and after, which may indicate a role in etiology, diagnosis and early therapeutic intervention [249]. Furthermore, metabolic reprogramming of striatal astrocytes in Huntington disease as an adaptation to decreased glucose supply leads to elevated ROS levels resulting in neuronal damage [250]. Modulation of astrocytic metabolic reprogramming shows promising results in controlling inflammation in neurodegenerative disorders [251]. Recent evidence indicates that loss of free fatty acid (FFA) degradation by astrocytic mitochondria results in lipid accumulation, reactive gliosis, and inhibition of lipid synthesis for structures including myelin. These changes may consequently produce metabolic shifts toward neuroinflammation, synaptic disruptions and microglial activation [252]. The mechanism of inducing AD by the genetic risk factor *APOE4* is also thought to include the disruption of lipid metabolism and related energetic and biosynthesis impairment [253].

Ketone body metabolism in both neurons and astrocytes changes with aging [223], and diet has been shown to act on brain aging markers such as network stability [254]. Furthermore, neuroprotective effects of ketosis induction [255,256,257,258] have been studied in epilepsy [259,260,261], GLUT1 deficiency [262,263], traumatic brain injury [264], migraine [265], neurodegenerative diseases [110,266,267,268], and neuroprogressive disorders, such as schizophrenia, bipolar disorder, and major depressive disorder [269]. Recently, β-hydroxybutyrate has also been studied as a potential therapy for aspartate/glutamate carrier (ARALAR/AGC1) deficiency [270]. However, it remains unclear whether the primary mechanism of ketogenic diet can be attributed to signaling and regulation or to the energy substrate role of ketones.

## 6. Sex-Dependent Brain Energy Decline in Aging and Neurodegeneration

In considering the human health implications of astrocytes in brain energy management in aging and neurodegeneration, sex and hormonal influences loom large. In women, menopause involves a decrease in estrogen production. Among its many powerful effects in reproductive cycles, estrogen also modulates brain glucose metabolism, as well as neurological functions that are surprisingly independent of reproduction [271]. Decreases in estrogen can result in reduced brain glucose usage, insulin resistance, mitochondrial insufficiency, myelin degeneration, and increased reliance on lipid metabolism [271,272]. For these and other reasons, the loss of estrogen could facilitate a path to AD [272]. In fact, women are about 50% more likely to die of AD than men and are at four times the risk of APOE4-linked dementia [273]. This heightened incidence is not explained by women living longer but rather might involve the removal of estrogen’s protective effects against amyloid-beta toxicity and mitochondrial decline after menopause [274].

During advanced aging, there are also specific susceptibilities between the sexes, for example regional metabolic differences, such as reduced metabolism in the caudate nucleus in males and the occipital lobe in females. While both sexes exhibit more rapid metabolic decline after 60, male brain metabolism is more affected after 70 [275]. Altogether it seems that while menopause presents greater dementia danger for susceptible women after menopause, in general they age more slowly metabolically than men, especially after reaching 70 years of age. More research is needed to clarify sex- and age-related differential diagnoses and treatments for brain metabolic disorders.

## 7. Summary and Outlook

For many decades, energy sources other than glucose remained in the shadows of this main brain fuel, but these alternatives are now increasingly seen as having both metabolic and signaling functions. Specific diets, such as ketogenic or caloric restriction, have been demonstrated to possess therapeutic properties, but the underlying molecular mechanisms remain largely unclear. We are still on the way to achieve a systematic understanding of different energy substrate roles in the brain and chart new routes on the biochemical network of neurometabolic interplay. At present, computational modeling is one of the best tools for bringing together disparate pieces of knowledge from otherwise not directly comparable studies. However, computational methods require experimental data for construction and validation. Better coordination between computational and experimental studies is a way to advance understanding of the crosstalk of brain signaling functions and metabolism.

We have investigated several energy substrates in this review: glucose, lactate, glycogen, ketones, and lipids. Each substrate performs multiple functions in the brain and serves as a preferred fuel in specific conditions (Table 2). Their common role in energy supply is accompanied by their involvement in different regulatory and signaling processes, which may highlight the importance of energy balance and an aspect of its tight regulation. Furthermore, these molecules contribute to neuromodulation, learning and memory, which reflect multiple parallel connections of neurometabolic and neurovascular coupling. As new aspects emerge, the relation between molecules and functions may appear as many-to-many, which emphasizes the importance of the highly synchronized and coordinated work of the brain as a molecular system.

Changes associated with all reviewed energy substrates are known to be implicated in aging and related disorders. However, these clues do not yet permit assembly of the full picture of aging and disease progression, and new links are yet to be discovered. Even though this review is limited to neurons and astrocytes, more fine-grained detail of their subtypes, other brain cell types, and brain region specificity should be considered to identify and develop more targeted therapies.

Due to the nature of biomedical research where multiple models are used to study various aspects of a system, the reader is cautioned that our summary may include results from experiments with various animal species, including humans, as well as in vivo or in vitro studies. A full dissection of the influence of experimental conditions in this context would be rather confusing and distracting. Unless some contradiction is clear from the experimental literature that should be pointed out in our paper, a deeper dive into areas of interest must take these details into consideration.

## 8. Conclusions and Perspectives

For decades astrocytes were considered to be second-tier cells of the brain, offering little more than glue-like support for neurons, as famously postulated by Virchow [287]. The historical perspective and early major studies of glia have been extensively reviewed by Fan and Agid [287]. However, the perception of glia has changed with the numerous advances in understanding the various functions of astrocytes that are critical to brain health. Across the main energy sources of the brain, astrocytes play fundamental metabolic and signaling roles, which are highly interdependent and condition-specific. Many of these roles are tightly related to brain energy metabolism, which is further implicated in brain aging and neurodegeneration. Astrocytes express a flexible adaptive phenotype, and their properties change dramatically between healthy and diseased brain states. Correspondingly, many gaps in our understanding of the broad repertoire of astrocytic functions remain in addition to what are certainly yet to be discovered functions.

## Figures and Tables

**Table 1:** Key differences in metabolism between neurons and astrocytes. The feature column includes those pathways, enzymes and transporters in which differences have been demonstrated between neurons and astrocytes. The neuron and astrocyte columns provide respective overviews of the cell-type specific features.

Feature	Neuron	Astrocyte	References
Glucose transporters	Glucose transporter 3 (GLUT3), Glucose transporter 4 (GLUT4, presynaptic terminals)	Glucose transporter 1 (GLUT1)	[17,18,19]
Glycogen storage and metabolism	Not present or low; age-dependent expression	Present	Astrocyte-specific: [20,21,22,23,24,25] Detected in neuron: [26,27] Presence in neuron is age-dependent: [28]
Predominant bioenergetic pathway	Oxidative phosphorylation (OXPHOS)	Glycolysis (however, some recent evidence suggests higher than previously thought share of mitochondrial adenosine triphosphate (ATP) in astrocytes [29,30])	[31]
Glycolytic rate	Lower	Higher	[32]
Hexokinase (HK)	Higher expression of HK1,2,3	Lower expression of HK1,2,3	[33]
6-phosphofructo-2-kinase/fructose-2,6-biphosphatase 3 (PFKFB3)	Low protein concentration (proteasomal degradation by anaphase-promoting complex/cyclosome (APC/C)-Cdh1)	High protein concentration (low APC/C-Cdh1 activity)	[32]
Pyruvate kinase M (PKM)	PKM1	PKM2	[34,35]
Glyoxalase system	Lower Glo1 and Glo2 activity than in astrocytes	Higher Glo1 (9.8 times) and Glo2 (2.5 times) activity than in neurons	[36,37]
Monocarboxylate transporters (MCTs)	MCT2 (high affinity for lactate)	MCT1,4 (MCT1,4 have lower affinity for lactate than MCT2)	[38]
Lactate dehydrogenase (LDH)	LDH1 (synapse)	Mostly LDH5	[38]
Pyruvate carboxylation	Less, potentially by malic enzyme	Pyruvate carboxylase, malic enzyme	[39,40,41,42,43]
Tricarboxylic acid cycle (TCA)	More active	Less active	[44]
Pyruvate dehydrogenase (PDH)	More active	Less active	[44]
Pyruvate dehydrogenase kinases (PDK)	Present	More active than in neurons; PDK4 expression is 30 time higher than in neurons	[34,45,46]
OXPHOS ETC (electron transport chain) Complex I	Mostly assembled into supercomplexes (lower reactive oxygen species (ROS) generation)	High ratio of free complex I (higher ROS generation)	[47]
MAS (malate–aspartate shuttle)	Particularly important	Extremely low activity	[48]
Antioxidant machinery, nuclear factor erythroid 2-related factor 2 (Nrf2)	Low	High	[49]
Glutathione (GSH) pool	Low	High	[50]
Pentose phosphate pathway (PPP)	Significant, very active, needed for redox balance	Four–five times higher activity than in the neurons; glucose-6-phosphate dehydrogenase (G6PD, rate limiting step) expressed higher than in the neurons	[32,51,52,53,54]
L-serine synthesis	Less	More	[31,55,56]
Folate metabolism, in particular, Aldehyde Dehydrogenase 1 Family Member L1 (ALDH1L1, cytosolyc) and L2 (ALDH1L2, mitochondrial)	Reduced	ALDH1L1 is astrocytic marker	[31,34,57,58]
Anaplerotics fluxes	Less	More	[59]
Fatty acid oxidation (FAO)	Almost not present	Present (about 20% of astrocytic needs [59]), enriched FAO enzymes	[34,60,61]

**Table 2:** Energy substrates preference of the brain in different conditions. Overall brain tissue-wide energy use preferences at different ages or conditions for four main substrates are shown.

Condition	Glucose	Lactate	Glycogen	Ketone Bodies
Embryonic	Used, levels are comparable to adult [276]	Used, higher levels of lactate than in adult [276]	Present in both neurons and astrocytes [26], but exact contribution as energy fuel is unclear and rather minor compared to glucose, lactate and ketone bodies	Used, higher levels of β-hydroxybutyrate than in adult [276]
Neonatal	Glucose supports 63% of the brain energy demands [277]	Lactate may serve as preferential fuel when concentrations are the same [277]	Lower levels than in embryonic and adult [276]	Ketones support 30% of the brain energy demands [277]
Childhood	Main source, higher rate of metabolism than in adult state [278]	Can be used but lower use than in neonatal period [277]	Secondary energy source used in specific conditions, such as those associated with increased energy demands	Higher capacity to use ketone bodies than in adult ages, but mostly associated with specific conditions like limited carbohydrates supply
Young, adult	Main source [279]	Can be used	Secondary energy source used in specific conditions, such as those associated with increased energy demands	Used but to less extent than glucose, unless under specific conditions
Aged	Decreased use [66]	Increased levels of brain lactate, shift in LDH A/B ratio [189]	Glycogen granules accumulation and decrease of glycogen metabolism [280]	Efficiently metabolized, potential therapeutic role [266]
Learning and memory	Used	Needed for memory consolidation [281]	Plays important role [281]	Used [242]
Sleep	Preferential fuel, but metabolism rates vary by sleep stages [282]	Decreased levels [283]	Replenishment of glycogen stores [284]	Increased use [285]
Arousal	Increased use	Exported from astrocytes [162]	Fuels lactate production [162]	Can be used
Alzheimer’s disease	Decreased use [66]	Exact role is unclear	Glycogen synthase kinase 3 (GSK3-beta) plays one of the key roles in AD pathology [66]	Used, ketogenic diet is suggested as therapeutic intervention [266,286]
